# Respiratory Disease in Adults during Pandemic (H1N1) 2009 Outbreak, Argentina

**DOI:** 10.3201/eid1512.091062

**Published:** 2009-12

**Authors:** Carlos Zala, Roberto Gonzalez

**Affiliations:** Hospital Central de San Isidro, Buenos Aires, Argentina

**Keywords:** Influenza, human, H1N1, virus, Latin America, Argentina, letter, expedited

**To the Editor:** We report a mild to moderate respiratory disease in patients seeking treatment for influenza-like illness (ILI) within the first 8 weeks of an outbreak of influenza A pandemic (H1N1) 2009 virus (pandemic [H1N1] 2009) infection in the Province of Buenos Aires. The first cases of pandemic (H1N1) 2009 in Argentina were reported in early May 2009 in travelers returning from Mexico and the United States. In mid-June, a sharp increase was reported in the number of patients with acute respiratory symptoms who were seeking treatment in emergency rooms. By July 9th, the Argentinean Ministry of Health had confirmed 2,677 cases and 82 deaths; most of those infected were residents of Buenos Aires and the surrounding area (*1*). When the World Health Organization raised the pandemic level to 6, >80% of circulating influenza A virus in Argentina was subtype H1N1 (*2*). At the Hospital Central de San Isidro, a tertiary hospital of 160 beds in the Province of Buenos Aires, initial clinical evaluation of patients with ILI symptoms included physical examination and, eventually, chest radiograph and pulse oximetry. Because diagnostic resources were limited, patients with ILI were eligible to receive oseltamivir with no prior sampling for respiratory pathogens. A standardized form was used for prescription and data collection, including demographic data, history of influenza vaccination, date symptoms began, and coexisting illnesses. From June 16 to July 5, a total of 2,135 patients with ILI were evaluated. The age of patients ranged from 14 to 82 years of age (median, 35 years); 854 patients (40%) were male. Because of lower respiratory disease, a total of 166 (8%) of 2,135 patients were admitted to the internal medicine ward (n = 139) or to the intensive care unit (n = 27). At admission, patients had >1 of the following: diffuse pulmonary infiltrates, room air oxygen saturation <95%, crackles on auscultation. Other clinical manifestations were not statistically different from those already reported in patients with pandemic (H1N1) 2009 in the United States, including cough, fever, and sore throat (*3*). The most common radiology pattern was basal, and bilateral interstitial infiltrates were consistent with primary viral pneumonia. Notably, some patients had clinical radiologic dissociation characterized by cough and pulmonary infiltrates in the absence of fever. Median time of hospitalization was 36 hours (range 1–25 days). No significant differences were observed between the groups of patients that were admitted versus outpatients in terms of age, sex, number of days from initiation of fever to first hospital visit, and history of influenza vaccination. A total of 163 (98%) of 166 patients admitted to the hospital during the observation period were discharged with no further complications.

Patients admitted to the hospital with pulmonary infiltrates were empirically treated with high dose oseltamivir (150 mg 2×/d) for 5 days, other antimicrobial drugs, and, eventually, steroids. In 2 patients, the respiratory disease progressed initially but they eventually recovered; 2 patients (1.2% of admissions to hospital) with acute respiratory failure died. Despite improvement in clinical symptoms at discharge, chest radiographs performed on a limited number of patients showed no substantial changes at 72–96 h after admission.

Clinical manifestations of pandemic (H1N1) 2009 have not yet been fully characterized. We observed a mild to moderate lower respiratory disease in ≈8% of consecutive patients with ILI during the current pandemic in Argentina. A more severe respiratory disease was observed in Mexico during the current pandemic (*4*) In contrast, early reports indicated that pandemic (H1N1) 2009 disease might be similar in severity to seasonal influenza (*3*). A lack of microbiologic confirmation may bias our observation. Because pulmonary infiltrates are uncommon in previously healthy persons with ILI, a simultaneous circulation of other respiratory pathogens may explain our observation. Furthermore, early empirical use of antimicrobial drugs could overshadow clinical features of bacterial pneumonia.

We observed an unexpectedly high rate of lower respiratory disease in adults with ILI during an outbreak of pandemic (H1N1) 2009 in Argentina. This finding suggests that a unique pattern of virulence, pulmonary tropism, or both may characterize the current influenza A (H1N1) infection, although we could not rule out co-infection with other viral or bacterial respiratory pathogens. Considering the evolving nature of influenza viruses, the wide clinical spectrum of pandemic (H1N1) 2009 should be further investigated.
